# Power beacon-assisted energy harvesting symbiotic radio networks: Outage performance

**DOI:** 10.1371/journal.pone.0313981

**Published:** 2025-02-05

**Authors:** Tran Cong Hung, Bui Vu Minh, Tan N. Nguyen, Miroslav Voznak

**Affiliations:** 1 Dean of School of Computer Science & Engineering, The SaiGon Internaltional University, Ho Chi Minh City, Vietnam; 2 Faculty of Engineering and Technology, Nguyen Tat Thanh University, Ho Chi Minh City, Vietnam; 3 Communication and Signal Processing Research Group, Faculty of Electrical and Electronics Engineering, Ton Duc Thang University, Ho Chi Minh City, Vietnam; 4 Faculty of Electrical Engineering and Computer Science, VSB-Technical University of Ostrava, Ostrava, Czechia; Northwestern Polytechnical University, CHINA

## Abstract

The evolution of next-generation Internet-of-Things (IoT) in recent years exhibits a unique segment that wireless communication paradigms are oriented towards not only improved spectral efficiency transmission but also energy efficiency. This paper addresses these critical issues by proposing a novel communication model, namely power beacon-assisted energy-harvesting symbiotic radio. In particular, the limited energy primary IoT source communicates with its destination by first harvesting energy from a dedicated power beacon and then performing information exchange, while the backscatter device communicates by exploiting the available radio frequency emitted by the primary IoT source. The destination uses successive interference cancellation mechanisms to decode both its received signals. To assess the performance quality of the proposed communication model, we theoretically derive the coexistence outage probability (COP) in terms of highly accurate expressions and upper-bound and lower-bound approximations. Subsequently, we carry out a series of numerical results to verify the developed theory frameworks on the one hand, and on the other hand, analyze the COP performance against the variations of system key parameters (transmit signal-to-noise ratio, the time-splitting coefficient, the energy conversion efficiency factor, the reflection coefficient, and the coexistent decoding threshold). Our numerical results demonstrate that the proposed communication model can potentially work well in practices with reliable communication over 90% (COP is less than 0.1). Additionally, it also demonstrates that optimizing the reflection coefficient at the backscatter device can facilitate achieving minimal COP performance.

## 1 Introduction

The next generation of wireless communication networks is envisioned to accommodate and support a wider array of mission-critical applications and services in response to the Internet-of-Things (IoT) era [1]. The involvement of several applications, such as smart homes, intelligent transportation, remote healthcare, and real-time monitoring services, has marked a major milestone in the evolution of IoT, where a huge number of IoT components, from mobile devices to sensors, with manifold quality-of-service (QoS) demands and energy profiles can be connected in an utmost effective manner [2]. However, this also puts additional pressure on managing spectral utilization and energy consumption.

In light of spectral efficiency enhancements, several approaches have been proposed in the literature to deal with this challenge. The first approach is to consider non-orthogonal multiple-access/rate-splitting [3] to enable multi-user transmission simultaneously within the power domain [4], instead of exploiting time and frequency resources. The second approach is to exploit cognitive radio (CR) to encourage spectrum sharing among licensed and unlicensed systems according to three models [5]: interweave, underlay, and overlay. Compared to interweave models, the underlay and overlay paradigms have received extensive attention from the research community [6–10]. On the other hand, there are also promising techniques in terms of communication to efficiently enhance spectrum utilization, such as full-duplex [11, 12], cooperative communication [13–15], device-to-device [16], two-way [17–20], terahertz [21, 22], and intelligent reflecting and(or) refractive surfaces [23, 24].

In light of energy efficiency improvements, two common energy harvesting (EH) methods in the literature are wireless power communication network (WPCN) and Simultaneous Wireless Information and Power Transfer (SWIPT), where WPCN separates the transmission of power and information transmission, with the representative harvest-and-use protocols [25]. Meanwhile, SWIPT [26] exploits the same radio frequency (RF) signal for transferring energy and information, with two typical protocols of time-switching and power-splitting. However, the communication raised by these active EH methods inherently yields relatively high power consumption as reported in [27–33], making it to be not favourable to large-scale IoT deployments. This raises the question of new sustainable alternatives for long-term deployment with low power consumption, prompting the emergence of the ambient backscatter communication (AmBAC) model [34]. AmBAC achieves its low-power goal by requiring a backscatter device to passively reflect and modulate a monotone sine wave based on the available RF signals. Instead of relying on the reader-sent continuous wave, the concept of symbiotic backscatter radio (SBR), a variation of AmBAC, was proposed to use the information-carrying signal sent by a non-power-constrained device to power a battery-free device [35–37].

Given the squeezed bandwidth resources for wireless communications and raised demands on low energy consumption, SBR has recently emerged as a promising communication paradigm to achieve these goals simultaneously for IoT networks [38], which is the main focus of this work. To reach this goal, symbiotic networks are expected to deploy IoT receivers capable of dual functions, i.e., they do not only receive signals from the primary transmitter but also the sensing signal emitted by backscatter devices (BD). And, recovering these signals are done by successive interference cancellation (SIC) mechanisms.

### 1.1 Related existing works

The existing body of work on SBR has seen remarkable advancements, beginning with early investigations where BDs rely on the incident primary signal to transmit its messages via the backscatter modulation without the need for active RF components [35]. This foundational work paved the way for a new SBR model, which allows the passive full-duplex BD to become parasitic during the active main transmission while improving the latency and spectral efficiency over the conventional broadcast channel model [36]. The exploration of cooperative paradigms introduced practical transmission schemes (commensal, parasitic, and competitive), with theoretical analyses under specific fading states [37]. Likewise, opportunistic commensal and parasitic mechanisms were proposed for pairing BD to not only strengthen the primary transmission but also improve the backscatter one [39].

Systematic reviews have been instrumental in addressing key challenges, such as enhancing backscattering links, achieving reliable communications through joint decoding, and leveraging reconfigurable intelligent surfaces (RIS) to capture primary transmitter signals [40]. Further research delved into non-cooperative and cooperative SBR systems with finite blocklength channel codes, deriving average achievable rates for both direct and backscatter links [41] or the approximated block-error rate combined with guidelines on how to get the simple asymptotic formulation [42].

Optimization efforts have focused on maximizing energy efficiency by jointly optimizing primary transmit power, reflection coefficients, and time-splitting coefficients in parasitic and commensal SBR systems with multiple BD deployments [43]. Advanced techniques have introduced multiple-input-multiple-output systems to enhance capacity rates [44] and cell-free massive multiple-input multiple-output systems to improve spectrum efficiency [45], supported by novel receiver designs for channel state information (CSI) acquisition [46].

Recent innovations have been particularly exciting, with hybrid backscatter and wireless power transfer schemes coupled with RIS to reduce energy consumption and maximize system throughput [47]. Symbiotic localization and AmBAC architectures have been developed to achieve mutual benefits in the sensing and communication stages [48]. Additionally, a novel symbiotic rate-splitting multiple-access communication paradigm was proposed to enhance spectral efficiency, supported by efficient beamforming design strategies [49].

These advancements collectively highlight the transformative potential of SBR in modern communication systems, paving the way for practical applications in IoT and beyond. The continuous evolution and innovation in this field underscore its significance and promise for future technological developments.

### 1.2 Motivations and contributions

This work aims to provide an efficient communication model that allows IoT networks with limited energy constraints can coexist together. In particular, IoT-limited energy networks can empower their communicating performance by exploiting either WPCN models [25] or SWIPT types [26]. As a common intuition from the literature that the use of a dedicated power beacon is widely adopted for energy-constrained IoT sources (i.e., WPCN) to enhance the energy-charging process [16, 30, 31], whereas backscatter communication networks live in the primary network and maintain their lifetime and communicating operations by following the SWIPT model without a dedicated power beacon [17, 27–29, 32, 33]. On the other hand, there are still situations where both the source and relaying node are energy limited that joint considerations of WPT and SWIPT [50] find their potential with different energy profiles. Nevertheless, the emergence of the IoT era in recent years promotes IoT to be developed towards multiple QoS requirements where IoT devices not only receive signals from one specific source of information but also others (i.e., sensing) for their diverse applications and services [38]. This is the time when symbiotic backscatter radio communication paradigms [51] have become a promising alternative in achieving spectrum utilization efficiently while facilitating the energy consumption issue. However, realizing the symbiotic backscatter radio communication paradigm mostly relies on the availability of the primary network deployment [35–37, 39–49]. To the best of the author’s knowledge, there are very rare investigations of a situation that which the primary network is constrained by an energy budget, particularly in the context of IoTs where the commonality of limited energy IoT sources is natural. This motivates us to develop an efficient communication model in response to this regard. The main contributions of the paper are the following:

- A power beacon-assisted energy harvesting symbiotic radio communication model is proposed by dedicating a power beacon station to empower the simultaneous communication of the primary IoT and backscatter networks that typically have limited energy constraints.
- The highly accurate expression for the coexistence outage probability (COP) has been derived in closed-form to deeply capture the system’s key parameters as well as the system performance.
- To deeply capture the most characteristic of the communication model, closed-form expressions for the lower-bound and upper-bound COP have been carried out. This is the basis for determining the diversity order and performance limits.
- We provide some simulation experiments to confirm the correctness of the derived COP expressions. Simultaneously, we also examine the influence of the indicators that is the *signal-to-noise ratio* (SNR), the time-splitting coefficient, the energy conversion efficiency factor, the reflection coefficient, and the coexistent decoding threshold. The simulation results not only verify our derived framework but also indicate the effectiveness of the proposed communication model in realizing the double objectives of enhanced spectral efficiency and improved energy consumption.

The remainder structure of this paper includes the system model description (Section 2), the performance analysis (Section 3), numerical results and discussions (Section 4), and the conclusion of the paper (Section 5).

## 2 System model description

**Fig 1:**
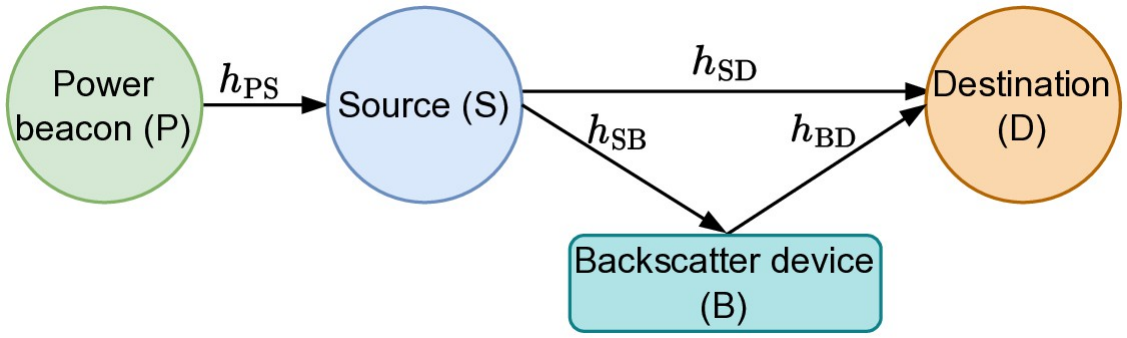
Illustration of the proposed power beacon-assisted energy harvesting symbiotic radio communication model.

As depicted in Fig 1, we propose a power beacon-assisted energy harvesting symbiotic radio communication model, including a dedicated power beacon (i.e., P), a source node (i.e., S), a backscatter device (i.e., B), and a destination (i.e., D). All nodes are assumed to be single antenna devices. In terms of communication, S with limited energy constrain communicates by harvesting energy from P and then using this energy for transmitting data information *s*(*t*) to D. Due to the nature of wireless broadcasting, B also receives this emitted signal simultaneously. Instead of decoding this signal, B exploits it as a carrier to convey its own symbol *c*(*t*) by changing its impedance and reflecting its modulation information back to D [36, 40, 48]. As a result, the received signals at D include two parts: 1) signal directly from S; and 2) reflecting signal from B. Due to multiplicative fading, D uses SIC to decode *s*(*t*) first and then *c*(*t*).

In this investigation, we assume that all links of communicating channels (as depicted in Fig 1) are available at D and follow quasi or static Rayleigh block fading, meaning that channels *h*_X_, with X ∈ {PS, SD, SB, BD}, keep stable within a considered block interval and only changes with the other ones. Thus, the probability density function (PDF) and the cumulative distribution function (CDF) of the channel gain |*h*_X_|^2^ can be expressed as

$$\begin{array}{c} f_{|h_{\text{X}}{|}^{2}}\left(x\right)=\lambda_{\text{X}}\;\text{exp}\;(-\lambda_{\text{X}}x),\;F_{|h_{\text{X}}{|}^{2}}\left(x\right)=1-\text{exp}(-\lambda_{\text{X}}x),\;x>0, \end{array}$$ (1)

where λ_X_ is the mean of |*h*_X_|^2^. To take into the simple pathloss model, λ_X_ can be modeled by $\lambda_{\text{X}}=d_{\text{X}}^{\chi }$, where *d*_X_ is the distance between two nodes and *χ* is the pathloss exponent.

Let’s consider a block duration *T* > 0 with two consecutive periods, where the first period *αT* is preserved for the energy harvesting process and the remaining period (1 − *α*)*T* is for data communication. During *αT*, the energy harvested from P at S can be given as in [27–30, 32, 33] as

$$\begin{array}{c} E_{S}=\alpha T\eta P_{P}{\left|h_{PS}\right|}^{2}, \end{array}$$ (2)

where *η* ∈ (0, 1) is the energy conversion efficiency and $P_{P}$ represents the transmit power of P. Thus, the transmit power of S used for data communication can be calculated as

$$\begin{array}{c} P_{S}=\frac{E_{S}}{(1-\alpha )T}=\frac{\alpha \eta TP_{P}{\left|h_{PS}\right|}^{2}}{(1-\alpha )T}=\kappa P_{P}{\left|h_{PS}\right|}^{2},\;\kappa ≜\alpha \eta /\left(1-\alpha \right). \end{array}$$ (3)

During the period time (1 − *α*)*T*, S transmits *s*(*t*) to D and B reflects the signal emitted by S to D with its own message *c*(*t*) satisfying $E\{|c\left(t\right){|}^{2}\}=1$. Accordingly, the received signal at D can be expressed as

$$\begin{array}{c} y_{D}=\sqrt{P_{S}}h_{SD}s\left(t\right)+\sqrt{\beta P_{S}}h_{SB}h_{BD}s\left(t\right)c\left(t\right)+n_{D}, \end{array}$$ (4)

where $n_{D}$ is the additive white Gaussian noise with zero mean and variance *N*_0_ and *β* is a reflection coefficient used to normalize *c*(*t*). Following the decoding rule *s*(*t*) → *c*(*t*), D decodes *s*(*t*) with the received *signal-to-interference-plus-noise ratio* (SINR) as

$$\begin{array}{c} {\bar{\gamma }}_{s}=\frac{P_{S}{\left|h_{SD}\right|}^{2}}{\beta P_{S}|h_{SB}{|}^{2}{\left|h_{BD}\right|}^{2}+N_{0}}=\frac{\kappa \Psi |h_{PS}{|}^{2}{\left|h_{SD}\right|}^{2}}{\beta \kappa \Psi |h_{PS}{|}^{2}|h_{SB}{|}^{2}{\left|h_{BD}\right|}^{2}+1}, \end{array}$$ (5)

where $\Psi =P_{P}/N_{0}$ is the average transmitting SNR. Leveraging the SIC technique, D can decode the backscatter message *c*(*t*) of B. Hence, the SNR of decoding *c*(*t*) at D can be expressed as

$$\begin{array}{c} {\bar{\gamma }}_{c}=\beta \kappa \Psi |h_{PS}{|}^{2}|h_{SB}{|}^{2}{\left|h_{BD}\right|}^{2}. \end{array}$$ (6)

Finally, the backscatter signal can be successfully decoded when *s*(*t*) and *c*(*t*) are perfectly decoded at D. Thus, the end-to-end received SINR and SNR at D can be claimed by

$$\begin{array}{c} {\bar{\gamma }}_{D}=\text{min}\left\{{\bar{\gamma }}_{s},{\bar{\gamma }}_{c}\right\}. \end{array}$$ (7)

Founding upon the above, we next move on evaluating the system’s COP performance.

## 3 Performance analysis

### 3.1 Outage definition

An outage event is defined as the probability that the received SNR/SINR at the receiving node falls below the predefined threshold [27–30]. Founding upon this definition, we denote by *γ*_th_ the predefined threshold for successfully decoding *s*(*t*) and *c*(*t*) at D. By the complementary probabilistic property, the coexistence outage probability (COP) at D can be defined as

$$\begin{array}{c} p_{\text{out}}=1-\text{Pr}[\gamma_{D}\geq \gamma_{\text{th}}]=1-\text{Pr}[\text{min}\left\{{\bar{\gamma }}_{s},{\bar{\gamma }}_{c}\right\}\geq \gamma_{\text{th}}]. \end{array}$$ (8)

### 3.2 Outage approximation analysis

By plugging the SINR in (5) and the SNR in (6) into (8), the COP of D can be rewritten as

$$\begin{array}{cc} p_{\text{out}} & =1-\text{Pr}[{\bar{\gamma }}_{s}\geq \gamma_{\text{th}},{\bar{\gamma }}_{c}\geq \gamma_{\text{th}}]\\ & =1-\text{Pr}[\frac{\kappa \Psi |h_{PS}{|}^{2}{\left|h_{SD}\right|}^{2}}{\beta \kappa \Psi |h_{PS}{|}^{2}|h_{SB}{|}^{2}{\left|h_{BD}\right|}^{2}+1}\geq \gamma_{\text{th}},\beta \kappa \Psi |h_{PS}{|}^{2}|h_{SB}{|}^{2}{\left|h_{BD}\right|}^{2}\geq \gamma_{\text{th}}]. \end{array}$$ (9)

From (9), the COP of D can be effectively evaluated by the following theorem.

**Theorem 1**. *The COP of* D *can be approximately derived as*

$$\begin{array}{cc} p_{\text{out}}≃1 & -\frac{\pi^{2}}{4K}\sum_{k=1}^{K}\sqrt{1-\phi_{k}^{2}}[\frac{\lambda_{PS}\lambda_{SB}\lambda_{BD}\gamma_{\text{th}}}{\beta \kappa \Psi \;\text{tan}([\phi_{k}+1]\pi /4)}\text{exp}(-\lambda_{PS}\;\text{tan}(\theta_{k})-\frac{\lambda_{SD}\gamma_{\text{th}}}{\kappa \Psi \;\text{tan}(\theta_{k})})\\ & \times \sum_{n=0}^{\infty }\frac{1}{n!}(-\frac{\lambda_{SD}\gamma_{\text{th}}\beta }{\lambda_{SB}\lambda_{BD}}{)}^{n}G_{1,3}^{3,0}(\frac{\lambda_{SB}\lambda_{BD}\gamma_{\text{th}}}{\beta \kappa \Psi \;\text{tan}(\theta_{k})}|\begin{array}{c} 0\\ -1,n,n \end{array})]{\text{sec}}^{2}\left(\phi_{k}\right), \end{array}$$ (10)

where *ϕ*_*k*_ = cos([2*k* − 1]*π*/2*K*), *θ*_*k*_ = [*ϕ*_*k*_ + 1]*π*/4, *K* is the accurate trade-off parameter, and $G_{p,q}^{m,n}(x|\begin{array}{c} a_{1},\cdots ,a_{p}\\ b_{1},\cdots ,b_{q} \end{array})$ is the Meijer function.

*Proof*. We begin the proof by first defining $X=|h_{PS}{|}^{2}$, $Y=|h_{SB}{|}^{2}{\left|h_{BD}\right|}^{2}$, and $Z=|h_{SD}{|}^{2}$. Herein, the distribution of *X* and *Z* follows (1), while the PDF of *Y* can be calculated as *f*_*Y*_(*y*) = ∂*F*_*Y*_(*y*)/∂*y*, where *F*_*Y*_(*y*) can be obtained as in [30] as

$$\begin{array}{c} F_{Y}\left(y\right)=1-2\sqrt{\lambda_{SB}\lambda_{BD}y}K_{1}\left(2\sqrt{\lambda_{SB}\lambda_{BD}y}\right), \end{array}$$ (11)

where $K_{1}\left(\cdot \right)$ is the modified Bessel function of second kind with 1-th order. By using the connection $\frac{d}{dx}\left(x^{v}K_{v}\left(x\right)\right)=-x^{v}K_{v-1}\left(x\right)$, we achieve

$$\begin{array}{c} f_{Y}\left(y\right)=2\lambda_{SB}\lambda_{BD}K_{0}\left(2\sqrt{\lambda_{SB}\lambda_{BD}y}\right). \end{array}$$ (12)

Next, having achieved the CDFs and PDF in hand, the COP of D in (9) can be rewritten as

$$\begin{array}{c} p_{\text{out}}=1-\int_{0}^{\infty }\underset{I}{\underset{︸}{\text{Pr}[\frac{\kappa \Psi xZ}{\beta \kappa \Psi xY+1}\geq \gamma_{\text{th}},\beta \kappa \Psi xY\geq \gamma_{\text{th}}]}}f_{X}\left(x\right)dx, \end{array}$$ (13)

where *I* can be reformulated as

$$\begin{array}{cc} I & =\text{Pr}[Z\geq \frac{\gamma_{\text{th}}\left(\beta \kappa \Psi xY+1\right)}{\kappa \Psi x},\beta \kappa \Psi xY\geq \gamma_{\text{th}}]\\ & =\int_{\frac{\gamma_{\text{th}}}{\beta \kappa \Psi x}}^{\infty }\text{Pr}[Z\geq \frac{\gamma_{\text{th}}\left(\beta \kappa \Psi xy+1\right)}{\kappa \Psi x}]f_{Y}\left(y\right)dy\\ & =\int_{\frac{\gamma_{\text{th}}}{\beta \kappa \Psi x}}^{\infty }[1-F_{Z}(\frac{\gamma_{\text{th}}\left(\beta \kappa \Psi xy+1\right)}{\kappa \Psi x})]f_{Y}\left(y\right)dy. \end{array}$$ (14)

To proceed, we substitute *F*_*Z*_(⋅) and *f*_*Y*_(⋅) into *I*, yielding the result

$$\begin{array}{cc} I & =\int_{\frac{\gamma_{\text{th}}}{\beta \kappa \Psi x}}^{\infty }\;\text{exp}(-\frac{\lambda_{SD}\gamma_{\text{th}}\left(\beta \kappa \Psi xy+1\right)}{\kappa \Psi x})2\lambda_{SB}\lambda_{BD}K_{0}\left(2\sqrt{\lambda_{SB}\lambda_{BD}y}\right)dy\\ & =2\lambda_{SB}\lambda_{BD}\;\text{exp}(-\frac{\lambda_{SD}\gamma_{\text{th}}}{\kappa \Psi x})\underset{J}{\underset{︸}{\int_{\frac{\gamma_{\text{th}}}{\beta \kappa \Psi x}}^{\infty }\text{exp}(-\lambda_{SD}\gamma_{\text{th}}\beta y)K_{0}\left(2\sqrt{\lambda_{SB}\lambda_{BD}y}\right)dy}}. \end{array}$$ (15)

To solve *J*, let’s denote *u* = *yβκ*Ψ*x*/*γ*th, which enables us reformulate *J* into

$$\begin{array}{c} J=\frac{\gamma_{\text{th}}}{\beta \kappa \Psi x}\int_{1}^{\infty }\text{exp}(-\frac{\lambda_{SD}\gamma_{\text{th}}^{2}}{\kappa \Psi x}u)K_{0}(2\sqrt{\frac{\lambda_{SB}\lambda_{BD}\gamma_{\text{th}}}{\beta \kappa \Psi x}u})du. \end{array}$$ (16)

Using Taylor series $\text{exp}\left(x\right)=\sum_{n=0}^{\infty }x^{n}/n!$ and [52, Eq: 6.592–4], we can rewrite the above as

$$\begin{array}{cc} J & =\frac{\gamma_{\text{th}}}{\beta \kappa \Psi x}\int_{1}^{\infty }\sum_{n=0}^{\infty }\frac{1}{n!}(-\frac{\lambda_{SD}\gamma_{\text{th}}^{2}}{\kappa \Psi x}{)}^{n}u^{n}K_{0}(2\sqrt{\frac{\lambda_{SB}\lambda_{BD}\gamma_{\text{th}}}{\beta \kappa \Psi x}u})du\\ & =\frac{\gamma_{\text{th}}}{\beta \kappa \Psi x}\sum_{n=0}^{\infty }\frac{1}{2n!}(-\frac{\lambda_{SD}\gamma_{\text{th}}\beta }{\lambda_{SB}\lambda_{BD}}{)}^{n}G_{1,3}^{3,0}(\frac{\lambda_{SB}\lambda_{BD}\gamma_{\text{th}}}{\beta \kappa \Psi x}|\begin{array}{c} 0\\ -1,n,n \end{array}). \end{array}$$ (17)

Now, by substituting *J* into (15), we obtain

$$\begin{array}{c} I=\frac{\lambda_{SB}\lambda_{BD}\gamma_{\text{th}}}{\beta \kappa \Psi }\sum_{n=0}^{\infty }\frac{1}{n!}(-\frac{\lambda_{SD}\gamma_{\text{th}}\beta }{\lambda_{SB}\lambda_{BD}}{)}^{n}\frac{1}{x}\text{exp}(-\frac{\lambda_{SD}\gamma_{\text{th}}}{\kappa \Psi x})G_{1,3}^{3,0}(\frac{\lambda_{SB}\lambda_{BD}\gamma_{\text{th}}}{\beta \kappa \Psi x}|\begin{array}{c} 0\\ -1,n,n \end{array}). \end{array}$$ (18)

Finally, having obtained *I* in hand gives us a chance to reformulate the COP of D in (13) as

$$\begin{array}{cc} p_{\text{out}}=1 & -\frac{\lambda_{PS}\lambda_{SB}\lambda_{BD}\gamma_{\text{th}}}{\beta \kappa \Psi }\sum_{n=0}^{\infty }\frac{1}{n!}(-\frac{\lambda_{SD}\gamma_{\text{th}}\beta }{\lambda_{SB}\lambda_{BD}}{)}^{n}\\ & \int_{0}^{\infty }\frac{1}{x}\text{exp}(-\lambda_{PS}x-\frac{\lambda_{SD}\gamma_{\text{th}}}{\kappa \Psi x})G_{1,3}^{3,0}(\frac{\lambda_{SB}\lambda_{BD}\gamma_{\text{th}}}{\beta \kappa \Psi x}|\begin{array}{c} 0\\ -1,n,n \end{array})dx. \end{array}$$ (19)

Unfortunately, the integral above is a tough task to find a closed-form expression. Therefore, we overcome this circumvent by applying the Gaussian-Chebyshev quadrature in [15], and after some algebraic steps, the COP of D in (19) can be approximately derived as in (10).

**Remark 1**. The result in (10) indicates that the COP at D can be indeed characterized by the key system parameters: 1) The fading parameters λ_X_ of links P → S, S → D, S → B, and B → D; 2) The splitting-time coefficient *α*, the energy conversion efficient *η*, and the transmit power of P; 3) the backscattering coefficient *β*; and 4) The predefined threshold *γ*_th_ for successfully decoding *s*(*t*) and *c*(*t*). This analytical solution allows us to evaluate the system performance without simulations since it only involves common and elementary functions that are built-in in popular software packages such as Matlab, Mathematical, and Maple.

### 3.3 Outage asymptotic analysis

This section aims to provide a useful mathematical framework for evaluating the system performance at high transmit SNR region, i.e., Ψ. Specifically, for sufficient large transmit SNR, we can regard Ψ → ∞ in terms of mathematical to approximate the COP in (9) as

$$\begin{array}{c} p_{\text{out}}\overset{\Psi \to \infty }{\approx }1-\text{Pr}[\frac{|h_{SD}{|}^{2}}{\beta |h_{SB}{|}^{2}{\left|h_{BD}\right|}^{2}}\geq \gamma_{\text{th}},\beta \kappa \Psi |h_{PS}{|}^{2}|h_{SB}{|}^{2}{\left|h_{BD}\right|}^{2}\geq \gamma_{\text{th}}]. \end{array}$$ (20)

On the foundation of (20), the asymptotic COP at D can be evaluated by the following lemma.

**Lemma 1**. *At high SNR, the COP of* D *can be upper and lower respectively bounded as*

$$p_{\text{out}}^{\text{up}}=1+\text{exp}(\frac{\lambda_{SB}\lambda_{BD}}{2\beta \gamma_{\text{th}}\lambda_{SD}})W_{-1,1/2}(\frac{\lambda_{SB}\lambda_{BD}}{\beta \gamma_{\text{th}}\lambda_{SD}})-G_{3,0}^{0,3}(\frac{\beta \kappa \Psi }{\lambda_{SB}\lambda_{BD}\gamma_{\text{th}}}|\begin{array}{c} 0,0,1\\ - \end{array}),\;$$ (21)

$$p_{\text{out}}^{\text{low}}=\text{exp}(\frac{\lambda_{SB}\lambda_{BD}}{2\beta \gamma_{\text{th}}\lambda_{SD}})W_{-1,1/2}(\frac{\lambda_{SB}\lambda_{BD}}{\beta \gamma_{\text{th}}\lambda_{SD}}),$$ (22)

where $W_{\cdot ,\cdot }(\cdot )$ is the Whittaker function.

*Proof*. We start the proof by first defining $X=|h_{PS}{|}^{2}$, $Y=|h_{SB}{|}^{2}{\left|h_{BD}\right|}^{2}$, and $Z=|h_{SD}{|}^{2}$ and then writing (20) as

$$\begin{array}{cc} p_{\text{out}} & =1-\text{Pr}[\frac{Z}{\beta Y}\geq \gamma_{\text{th}},\beta \kappa \Psi XY\geq \gamma_{\text{th}}]\\ & =1-\int_{x=0}^{\infty }\int_{z=0}^{\infty }\text{Pr}[\frac{z}{\beta \gamma_{\text{th}}}\geq Y\geq \frac{\gamma_{\text{th}}}{\beta \kappa \Psi x}]f_{Z}\left(z\right)dzf_{X}\left(x\right)dx\\ & =1-\int_{x=0}^{\infty }\{\underset{Q}{\underset{︸}{\int_{z=0}^{\infty }[F_{Y}(\frac{z}{\beta \gamma_{\text{th}}})-F_{Y}(\frac{\gamma_{\text{th}}}{\beta \kappa \Psi x})]f_{Z}\left(z\right)dz}}\}f_{X}\left(x\right)dx. \end{array}$$ (23)

Next, we make use of (11) for the inner integral above to get the following

$$\begin{array}{cc} Q & =1-F_{Y}(\frac{\gamma_{\text{th}}}{\beta \kappa \Psi x})-\int_{0}^{\infty }2\sqrt{\frac{\lambda_{SB}\lambda_{BD}z}{\beta \gamma_{\text{th}}}}K_{1}(2\sqrt{\frac{\lambda_{SB}\lambda_{BD}z}{\beta \gamma_{\text{th}}}})\lambda_{SD}\;\text{exp}\left(-\lambda_{SD}z\right)dz\\ & =1-F_{Y}(\frac{\gamma_{\text{th}}}{\beta \kappa \Psi x})-\text{exp}(\frac{\lambda_{SB}\lambda_{BD}}{2\beta \gamma_{\text{th}}\lambda_{SD}})W_{-1,1/2}(\frac{\lambda_{SB}\lambda_{BD}}{2\beta \gamma_{\text{th}}\lambda_{SD}}), \end{array}$$ (24)

where the second step above is derived thanks to the help of [52, Eq: 6.643.3]. Now, we inject *Q* into (23) to arrive at the following

$$\begin{array}{cc} p_{\text{out}} & =1-\int_{x=0}^{\infty }\{1-F_{Y}(\frac{\gamma_{\text{th}}}{\beta \kappa \Psi x})-\text{exp}(\frac{\lambda_{SB}\lambda_{BD}}{2\beta \gamma_{\text{th}}\lambda_{SD}})W_{-1,1/2}(\frac{\lambda_{SB}\lambda_{BD}}{2\beta \gamma_{\text{th}}\lambda_{SD}})\}f_{X}\left(x\right)dx\\ & =1+\text{exp}(\frac{\lambda_{SB}\lambda_{BD}}{2\beta \gamma_{\text{th}}\lambda_{SD}})W_{-1,1/2}(\frac{\lambda_{SB}\lambda_{BD}}{2\beta \gamma_{\text{th}}\lambda_{SD}})\\ & \;-\lambda_{PS}\int_{0}^{\infty }\text{exp}\left(-\lambda_{PS}x\right)2\sqrt{\frac{\lambda_{SB}\lambda_{BD}\gamma_{\text{th}}}{\beta \kappa \Psi x}}K_{1}(2\sqrt{\frac{\lambda_{SB}\lambda_{BD}\gamma_{\text{th}}}{\beta \kappa \Psi x}})dx. \end{array}$$ (25)

Continuously, we use [52, Eqs: 9.34.3 and 9.31.2] for the COP of D in (25), which leads to

$$\begin{array}{cc} p_{\text{out}} & =1+\text{exp}(\frac{\lambda_{SB}\lambda_{BD}}{2\beta \gamma_{\text{th}}\lambda_{SD}})W_{-1,1/2}(\frac{\lambda_{SB}\lambda_{BD}}{2\beta \gamma_{\text{th}}\lambda_{SD}})\\ & \;-\lambda_{PS}\int_{0}^{\infty }\text{exp}\left(-\lambda_{PS}x\right)G_{2,0}^{0,2}(\frac{\beta \kappa \Psi x}{\lambda_{SB}\lambda_{BD}\gamma_{\text{th}}}|\begin{array}{c} 0,1\\ - \end{array})dx. \end{array}$$ (26)

Now, making use of [52, Eq: 7.831.5], we obtain the upper bound for the COP of D. Meanwhile, we make use of the approximation $K_{1}\left(x\right)∼1/x$ as *x* → 0 in [31] to rewrite (25) as

$$\begin{array}{c} p_{\text{out}}=1+\text{exp}(\frac{\lambda_{SB}\lambda_{BD}}{2\beta \gamma_{\text{th}}\lambda_{SD}})W_{-1,1/2}(\frac{\lambda_{SB}\lambda_{BD}}{2\beta \gamma_{\text{th}}\lambda_{SD}})-\lambda_{PS}\int_{0}^{\infty }\text{exp}\left(-\lambda_{PS}x\right)dx. \end{array}$$ (27)

By simplifications, we obtain the lower bound for the COP of D.

**Remark 2**. It follows from (21) that the upper bound of the COP of D is a function of Ψ, meaning that increasing Ψ improves the COP of D. However, the lower bound in (22) discloses that the COP of D is indeed an independent function of Ψ. That is to say, the COP at D does not improve with an increase in Ψ at a high SNR region (i.e., there exists a COP floor), and this confirms the zero-diversity order of D.

## 4 Numerical results and discussions

In this section, we employ the simulation results to verify the mathematical framework developed in the previous section, i.e., Eqs (10), (21) and (22). Herein, the Monte-Carlo simulation approach is used for simulation results by generating 10^3^ channel realizations for complex random Rayleigh variables *h*_X_, then putting them into the SINR in (5) and SNR in (6) to evaluate the end-to-end outcome in (7), and finally count on the COP based on the definition (8) by averaging outage event samples (i.e., dividing the total number of obtained outage event for the total test samples 10^3^). Besides, we also analyze how the COP of D changes with the transmit SNR Ψ, the time-splitting coefficient *α*, the reflection coefficient *β*, the energy conversion efficiency factor *η*, and the predefined decoding threshold $\gamma_{\text{th}}$. Unless others mention specific, we set $\lambda_{PS}=\lambda_{SB}=0.5$, $\lambda_{BD}=\lambda_{SD}=1$, *η* = 0.8, $\beta =\alpha =\gamma_{\text{th}}=0.25$, *K* = 50, and 10^3^ channel realizations.

**Fig 2:**
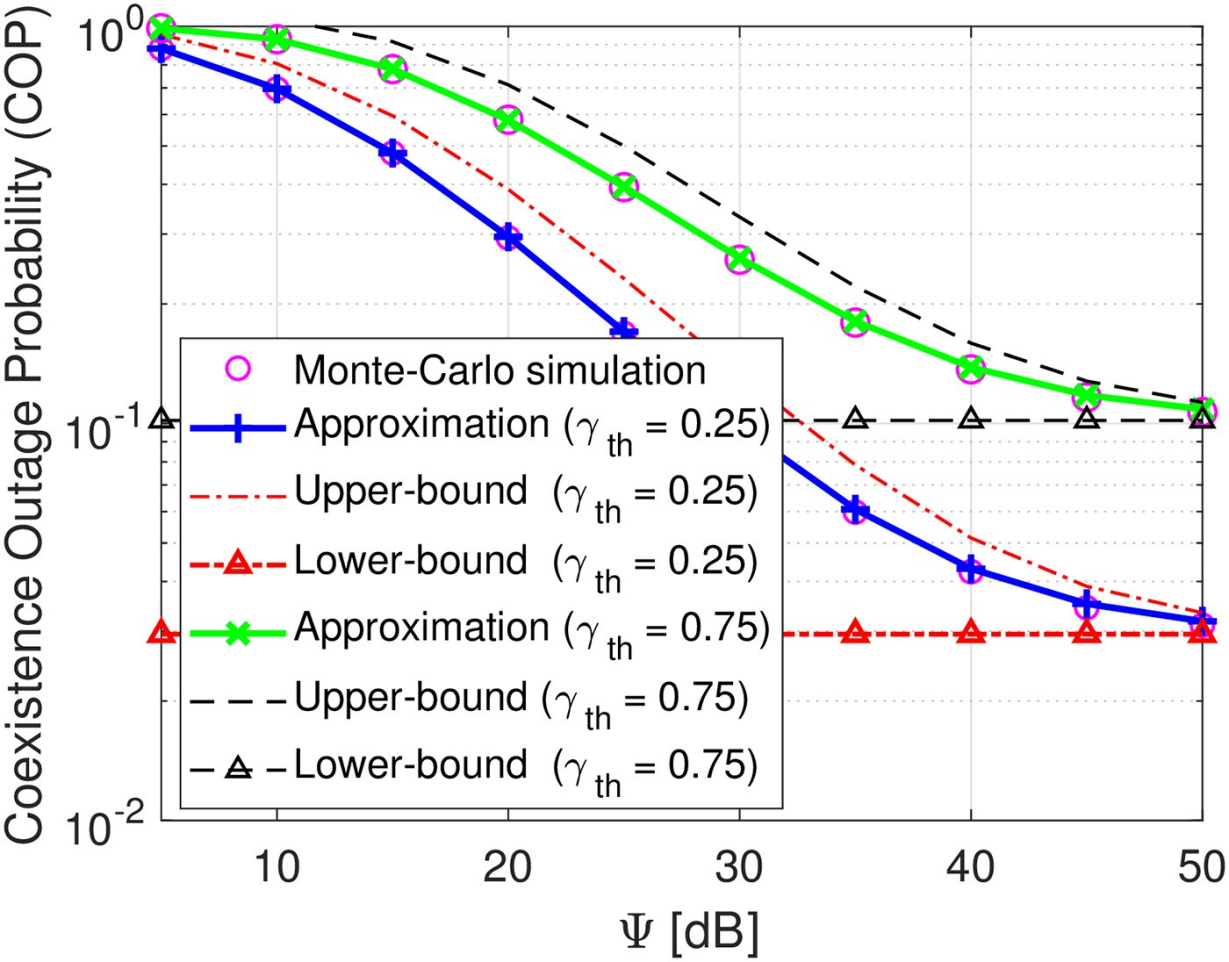
COP versus Ψ in dB under settings of the predefined decoding threshold *γ*_th_ = 0.25, 1.

**Fig 3:**
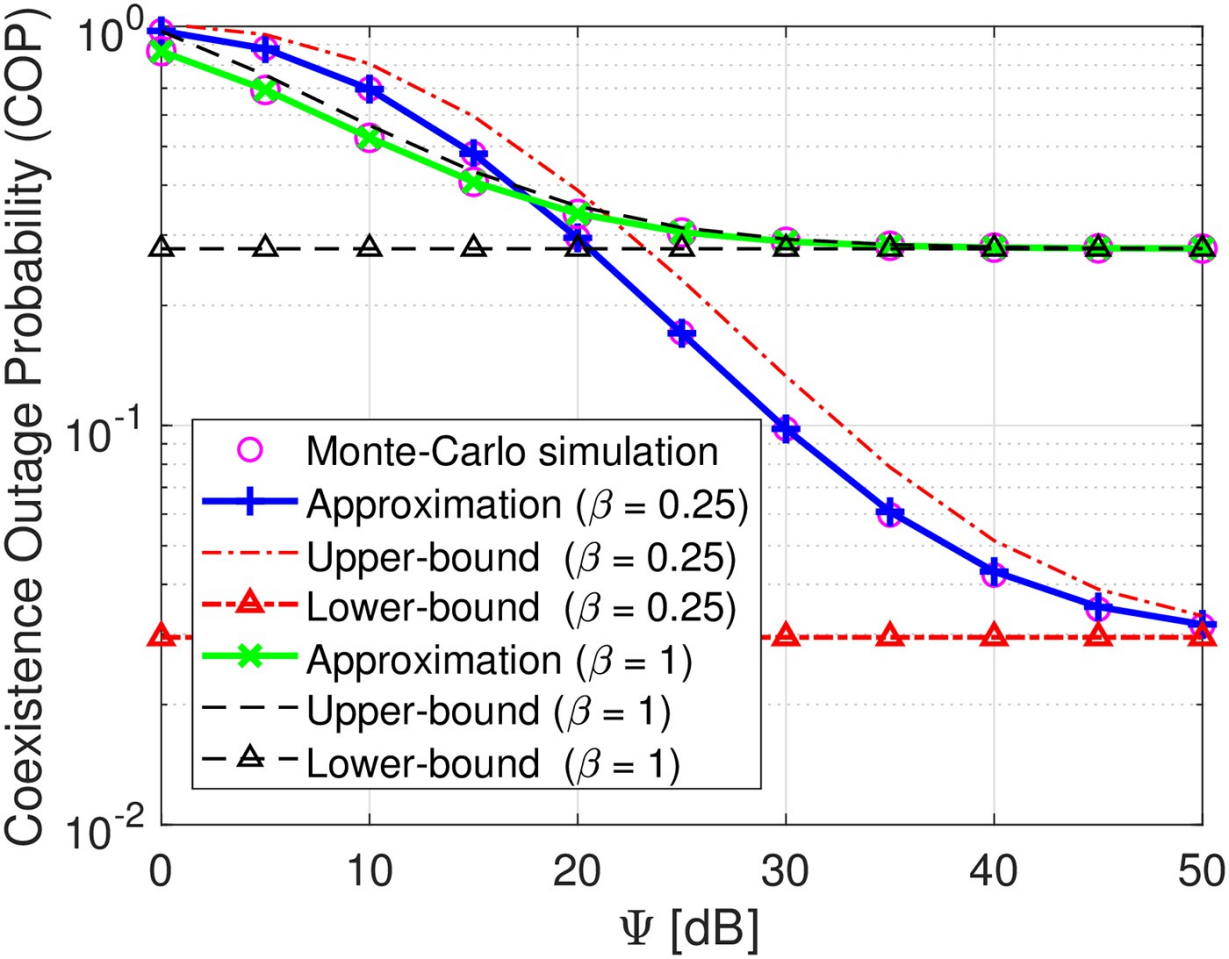
COP versus Ψ in dB under settings of the reflection coefficient *β* = 0.25, 1.

**Fig 4:**
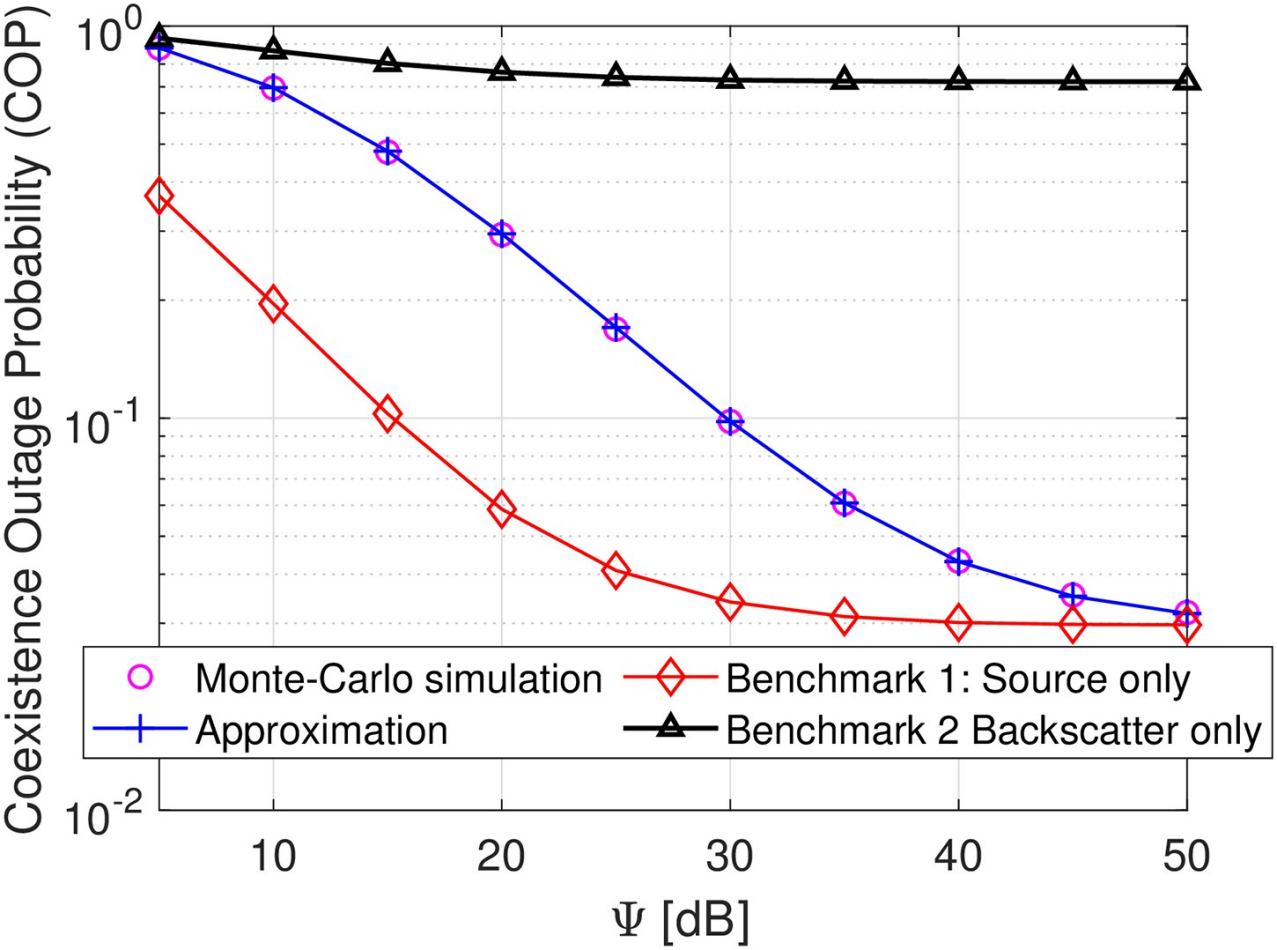
COP versus Ψ in dB under settings of *β* = *α* = *γ*_th_ = 0.25.

Figs 2–4 plot the COP of transmit SNR D as a function of Ψ. As observed, the Monte-Carlo simulation markers (i.e., magenta circle) align well with the theoretical approximated analyses, confirming the correctness of the developed derivation. Besides, it is also shown that as the transmit SNR Ψ increases, the COPs of D tend to decrease at low and moderate SNR and approach the upper and lower bound at high SNR, verifying the correctness of the theoretical derivations in Eqs (21) and (22) as well as our intuition in Remark 2. This is because when SNR changes from the lower to moderate regions, the decoding capabilities of *s*(*t*) and *c*(*t*) are both enhanced, thereby improving the COP of D. Conversely, at high SNR, the COP of decoding *s*(*t*) and *c*(*t*) is now highly dominated by a constant SINR ${\bar{\gamma }}_{s}$ since the fact that ${\bar{\gamma }}_{c}\to \infty$, yielding no improvement on the COP of D. With the same transmit SNR, Fig 2 demonstrates that the COP of D decreases as *γ*_th_ increases from 0.5 to 1. This is attributable to the fact that a higher *γ*_th_ is required, and the probability of successful decoding *s*(*t*) and *c*(*t*) is decreased, thereby the COP of D is higher. Similarly, Fig 3 reveals that increasing the reflection coefficient *β* of B also increases the COP of D. This is because a higher *β* increases the interference in the denominator of ${\bar{\gamma }}_{s}$, thereby decreasing the probability of successfully decoding *s*(*t*). The COP of D is increased consequently. In Fig 4, we plot the COP performance of the proposed system versus two benchmark schemes. The first benchmark scheme considers the destination with source information decoding only, while the second one assumes the destination with backscattering signal decoding only. As observed, when the destination functions with only source information decoding, its outage probability improves significantly at low and moderate values of Ψ and becomes saturated at high Ψ. This is because, at low levels of Ψ, the signal transmitted by the source is stronger than the backscattering interference. Conversely, when the destination operates with only backscattering signal decoding, its reception suffers from strong interference emitted by the source node, increasing the outage probability. Meanwhile, the proposed symbiotic system (which performs dual functions of source and backscatter decoding) achieves better outage performance than benchmark scheme 2, although it is slightly reduced compared to benchmark scheme 1. Therefore, considering the benefits of dual functions, our proposed symbiotic scheme shows significant promise for the future development of wireless communication systems. To capture more useful information for this proposed system design, let us study key system parameters’ impacts.

**Fig 5:**
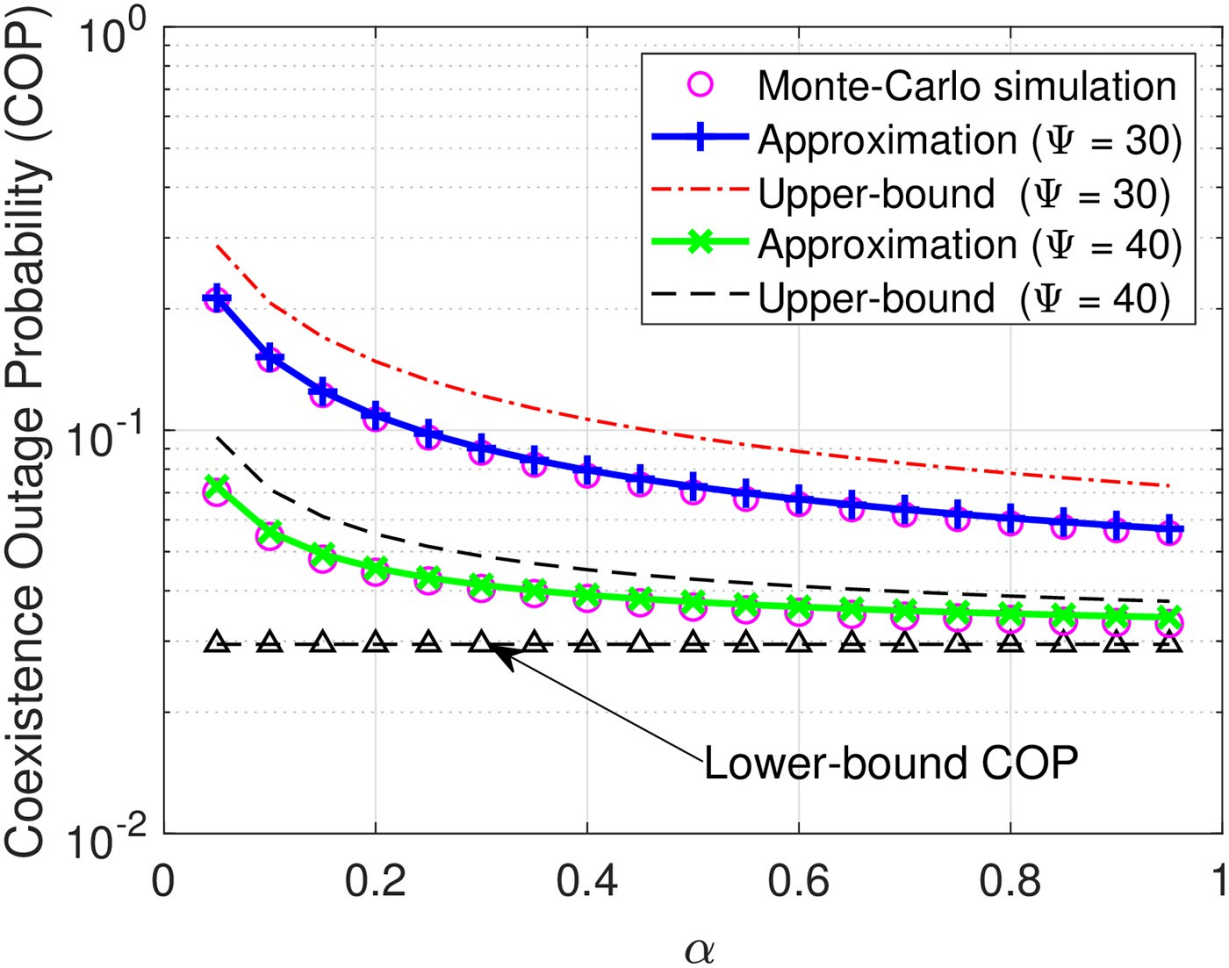
COP versus *α* under settings of the transmit SNR Ψ = 30, 40 in dB.

**Fig 6:**
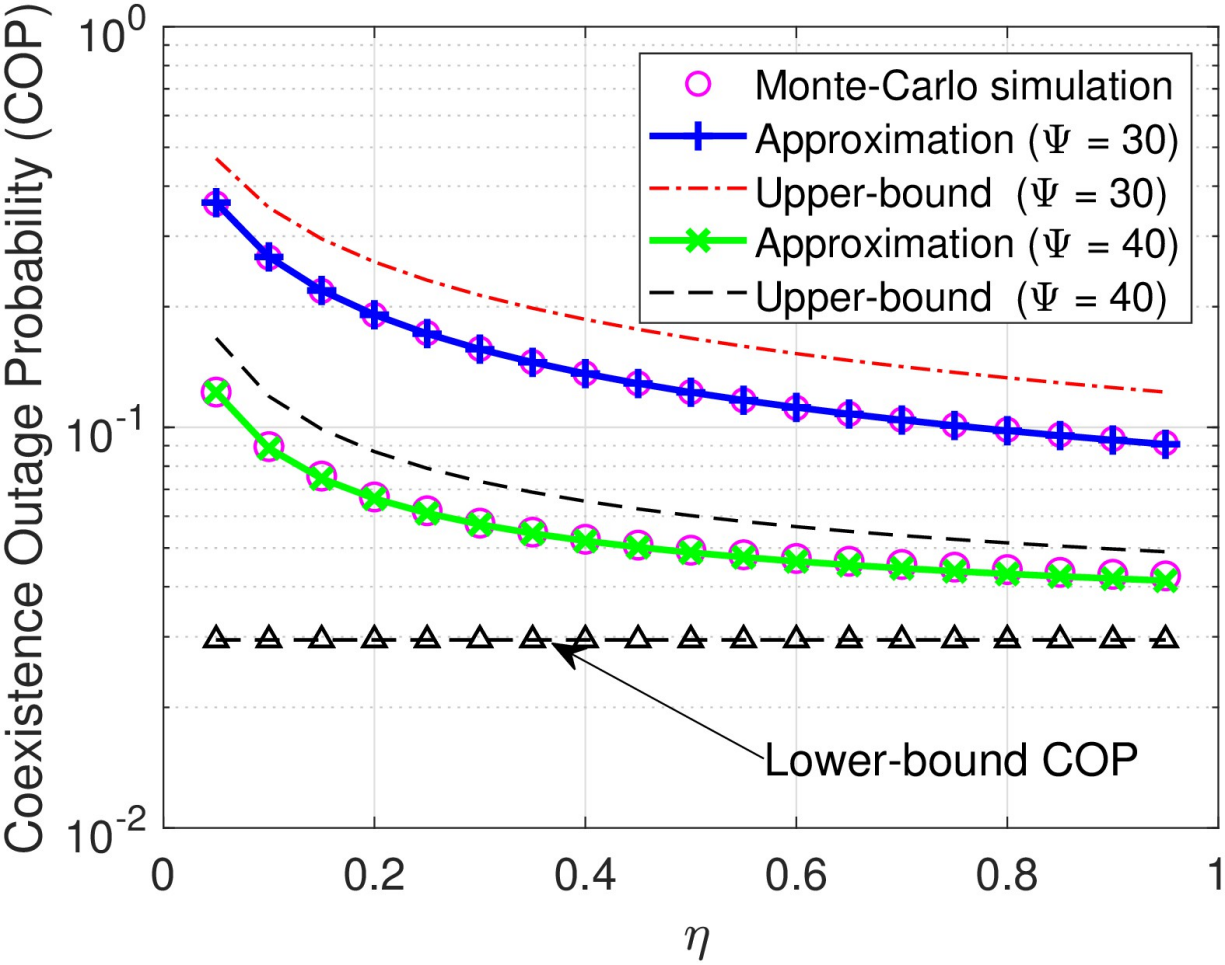
COP versus *η* under settings of the transmit SNR Ψ = 30, 40 dB.

Next, we plot the COP of D in relation to the time-splitting coefficient *α* in Fig 5 and the energy conversion efficiency factor *η* in Fig 6. It can be seen from Fig 5 that under the same SNR Ψ, the COP of D decreases rapidly as *α* increases from 0 to 0.6 and then converges lowly in the remaining range. This reason for the first phenomenon is that the higher the time allocation for the energy harvesting process, the larger the transmit power for the information. As for the second phenomenon, it is because increasing *α* for the energy harvesting process results in a lower time allocation, i.e., (1 − *α*)*T*, for the information transmission, thereby leading to a relatively low improved COP. The same intuition can be found in Fig 5, where the COP of D rapidly decreases as *η* varies from 0 to 0.8 but lowly changes with the remaining range of *η*.

**Fig 7:**
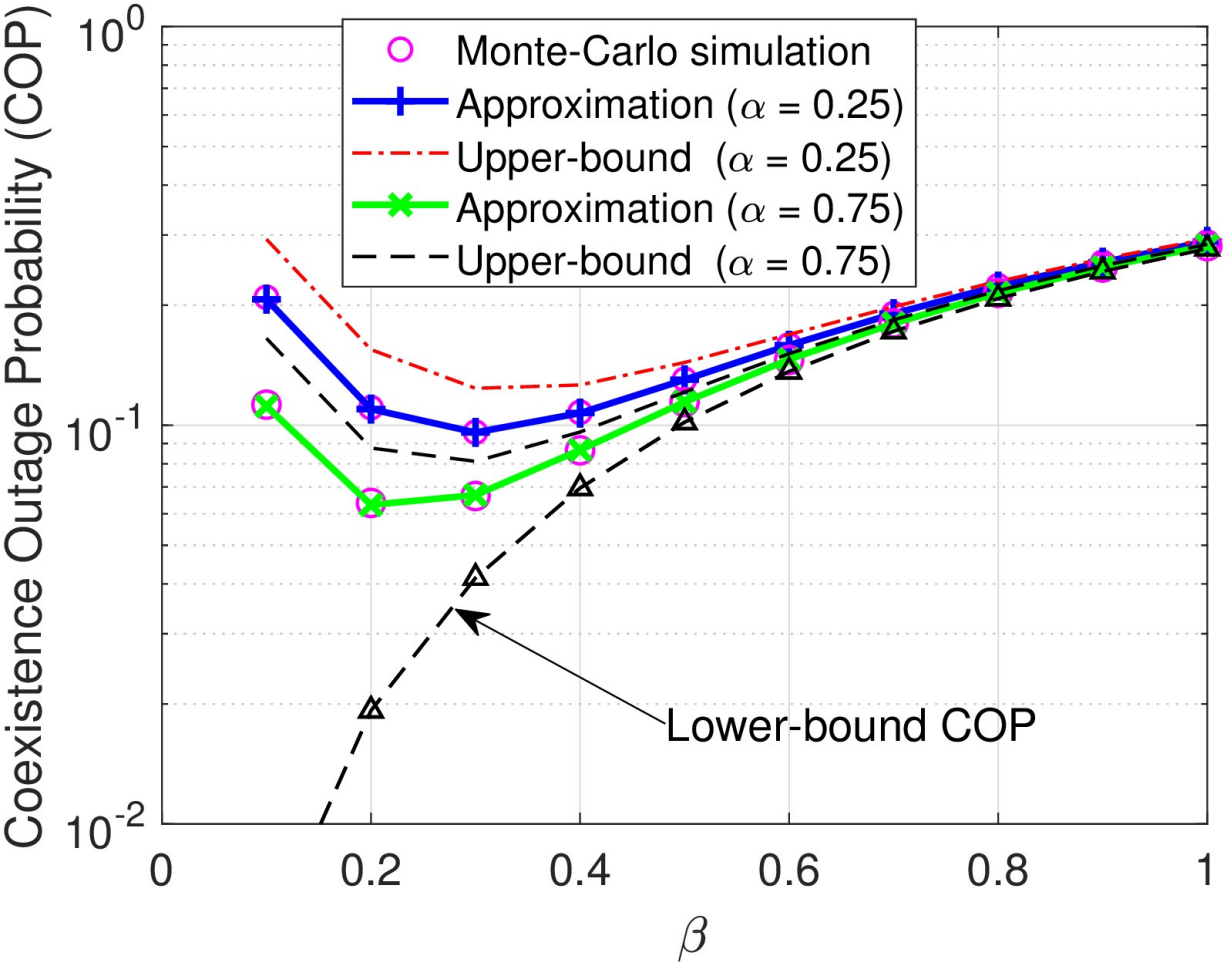
COP versus *β* under settings of the time-splitting coefficient *α* = 0.25, 0.75.

Fig 7 illustrates the variation of the reflection coefficient *β* of B for different the time-splitting coefficient *α* at Ψ = 40 dB. Fig 7 shows that the COP of D decreases with *β* varying from 0 to 0.3 and then linearly increases. This is because a smaller value *β* results in better decoding *c*(*t*); however, the more pronounced increment of *β* causes a higher interference for decoding *s*(*t*), leading to an increase in the COP of D. Clearly, there is a trade-off in setting *β* to achieve the optimal COP performance of D, where finding the optimal value *β* can be effectively obtained using the Golden-section search method [31].

## 5 Conclusion

In this paper, the performance of wireless-powered symbiotic radio systems was investigated, where a limited-energy source harvests energy from the dedicated power beacon to communicate with its destination and the destination exploits SIC to not only decode its primary signal but also the backscattering signal. Aiming to evaluate the system performance, the approximate expressions of COP for the destination and the lower and upper bound COP were also derived. Furthermore, these developed mathematical frameworks allow us to directly evaluate the effects of the transmit SNR, the time-splitting coefficient, the reflection coefficient, the energy conversion efficiency factor, and the predefined decoding threshold on the COP of the destination. Numerical results showed that our developed mathematical framework could accurately predict the simulation results. Additionally, the COP performance reflected and confirmed that exploiting the proposed model could be well realized in practice for symbiotic intention due to an error decoding probability of less than 10 percent. Moreover, it was also revealed that the COP is a convex function of the reflection coefficient shown by Fig 7, where the COP tends to decrease and then increase with the increase of the reflection coefficient. Therefore, by optimizing the reflection coefficient, the COP performance could be improved. Furthermore, it is a promising approach to ensure symbiotic communication as well as realize the goal of net zero for future green communication. Finally, analyzing the COP performance in consideration of more general channel models, imperfect channel estimation, and multi-antenna setups can be an exciting avenue for future investigation.

## Supporting information

S1 File (ZIP)

S2 File (ZIP)
