# Supplementary figures and images for: Power beacon-assisted energy harvesting symbiotic radio networks: Outage performance
Source: PLoS One. 2025 Feb 5;20(2):e0313981. doi: 10.1371/journal.pone.0313981 (PMC11798487; doi:10.1371/journal.pone.0313981)

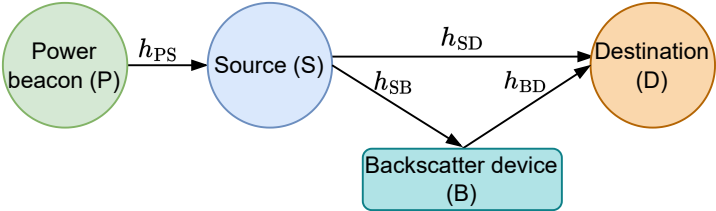

Supplement: S2 File — (ZIP) [file pone.0313981.s002.zip › Photo/figure1.pdf]
